# Supplementary material for: Association between DNA methylation levels in brain tissue and late-life depression in community-based participants
Source: Transl Psychiatry. 2020 Jul 30;10:262. doi: 10.1038/s41398-020-00948-6 (PMC7393126; doi:10.1038/s41398-020-00948-6)
Supplement: Supplementary file 1 — Supplementary material [file 41398_2020_948_MOESM1_ESM.docx]

**Supplementary Material**

**Association between DNA Methylation Levels in Brain Tissue and Late-Life Depression**

**in Community-Based Participants**

Anke Hüls, PhD^1,2^, Chloe Robins, PhD^3^, Karen N. Conneely, PhD^2^, Philip L. De Jager, MD, PhD^4,5^, David A. Bennett, MD^6^, Michael P. Epstein, PhD^2^, Thomas S. Wingo, MD^2,3^, Aliza P. Wingo, MD^7,8^

^1^Department of Epidemiology and Gangarosa Department of Environmental Health, Rollins School of Public Health, Emory University, Atlanta, Georgia, USA

^2^ Department of Human Genetics, Emory University, Atlanta, Georgia, USA

^3^ Department of Neurology, Emory University School of Medicine, Atlanta, GA, US

^4^ Cell Circuits Program, Broad Institute, Cambridge, MA, USA

^5^ Center for Translational and Computational Neuroimmunology, Department of Neurology, Columbia University Medical Center, New York, NY, USA

^6^ Rush Alzheimer’s Disease Center, Rush University Medical Center, Chicago, Illinois, USA

^7^ Division of Mental Health, Atlanta VA Medical Center, Decatur, GA, USA

^8^ Department of Psychiatry, Emory University School of Medicine, Atlanta, GA, US

**
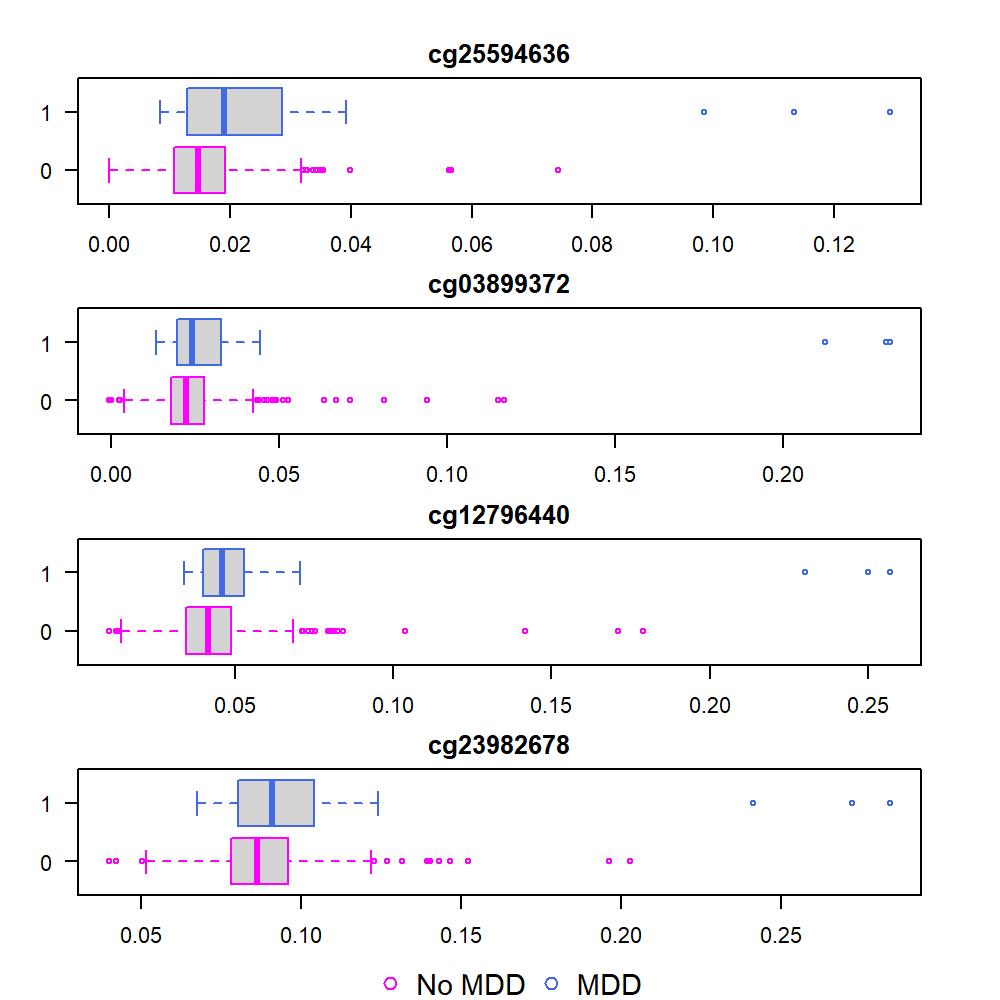
**

**Figure S1.** Distribution of DNA methylation beta values of the four most significant CpG sites in the YOD1 locus stratified by diagnosis of MDD at baseline visit. 1 indicates cases with major depressive disorder and 0 refers to controls.

**
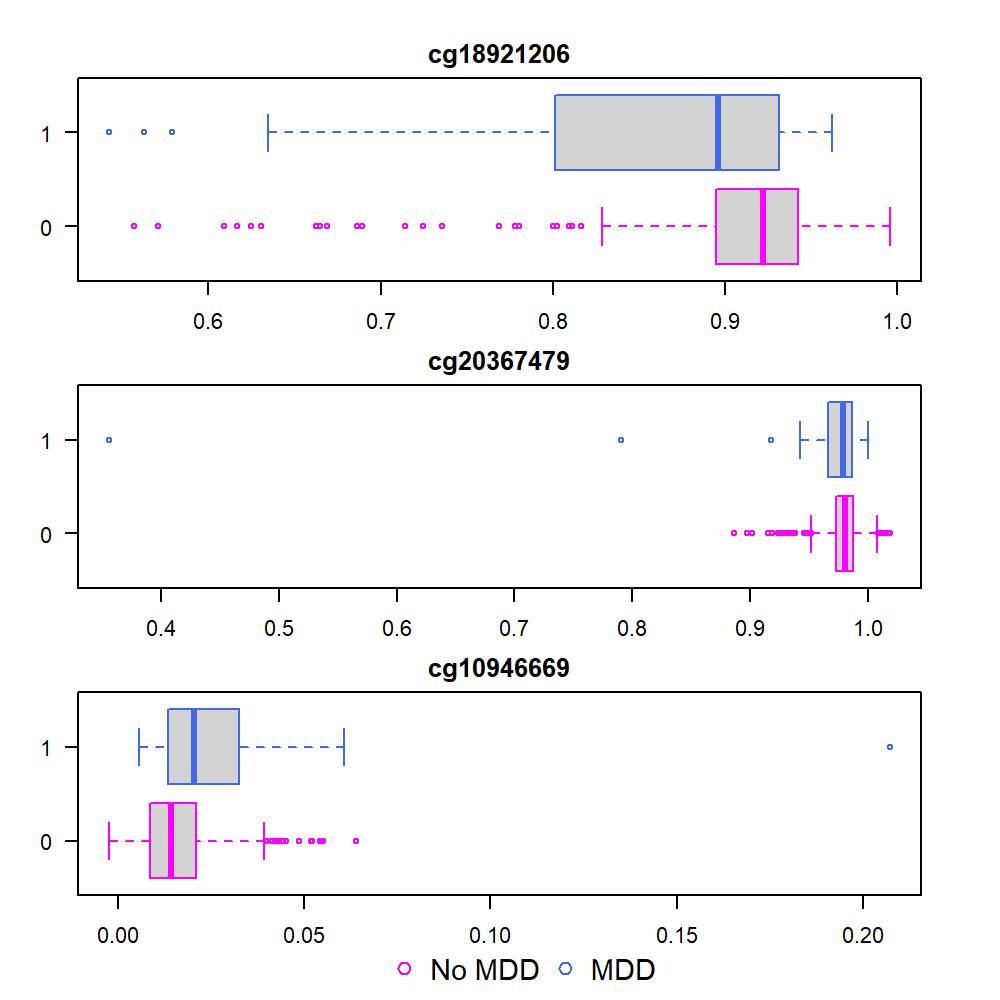
**

**Figure S2.** Distribution of DNA methylation beta values of the four most significant CpG sites in the *UGT8, FNDC3B* and *SLIT2* loci stratified by diagnosis of MDD at baseline visit. 1 indicates cases with major depressive disorder and 0 refers to controls.

| **Table S2.** Associations between DNA methylation and MDD tested with limma (main analysis), linear regression and permutation test. | | | | | | | | |
| --- | --- | --- | --- | --- | --- | --- | --- | --- |
|  |  |  |  | **Limma** | | **Linear regression** | | **Permutation test^#^** |
| **cpg** | **chr** | **position** | **Nearest Gene** | **Δ beta** | **p-value** | **Δ beta** | **p-value** | **p-value** |
| cg25594636 | 1 | 207224388 | *YOD1* | 0.013 | 2.55E-11 | 0.013 | 2.35E-11 | 4.00E-06 |
| cg03899372 | 1 | 207224102 | *YOD1* | 0.020 | 3.12E-09 | 0.020 | 3.17E-09 | 9.40E-05 |
| cg12796440 | 1 | 207224331 | *YOD1* | 0.022 | 1.51E-08 | 0.022 | 1.54E-08 | 4.50E-05 |
| cg18921206 | 4 | 115320920 | *UGT8* | -0.067 | 1.75E-08 | -0.067 | 1.84E-08 | 7.00E-06 |
| cg20367479 | 3 | 171873675 | *FNDC3B* | -0.032 | 4.97E-08 | -0.032 | 5.16E-08 | 0.0003 |
| cg23982678 | 1 | 207224227 | *YOD1* | 0.021 | 7.94E-08 | 0.021 | 8.12E-08 | 3.40E-05 |
| cg10946669 | 4 | 20253130 | *SLIT2* | 0.013 | 8.01E-08 | 0.013 | 7.91E-08 | 3.90E-05 |
| Bonferroni threshold: 1.22 x 10^-07^. Adjusted for age at death, sex, PMI, neuron proportions and the first three principal components from the genotype data. **^#^**adjusted linear regression with p-value from 1,000,000 permutations  Δ beta: This coefficient represents the mean difference of DNA methylation beta values between participants with and without MDD. Negative coefficients refer to smaller mean DNA methylation beta values in participants with MDD and positive coefficients refer to larger mean DNA methylation beta values in participants with MDD. | | | | | | | | |

**
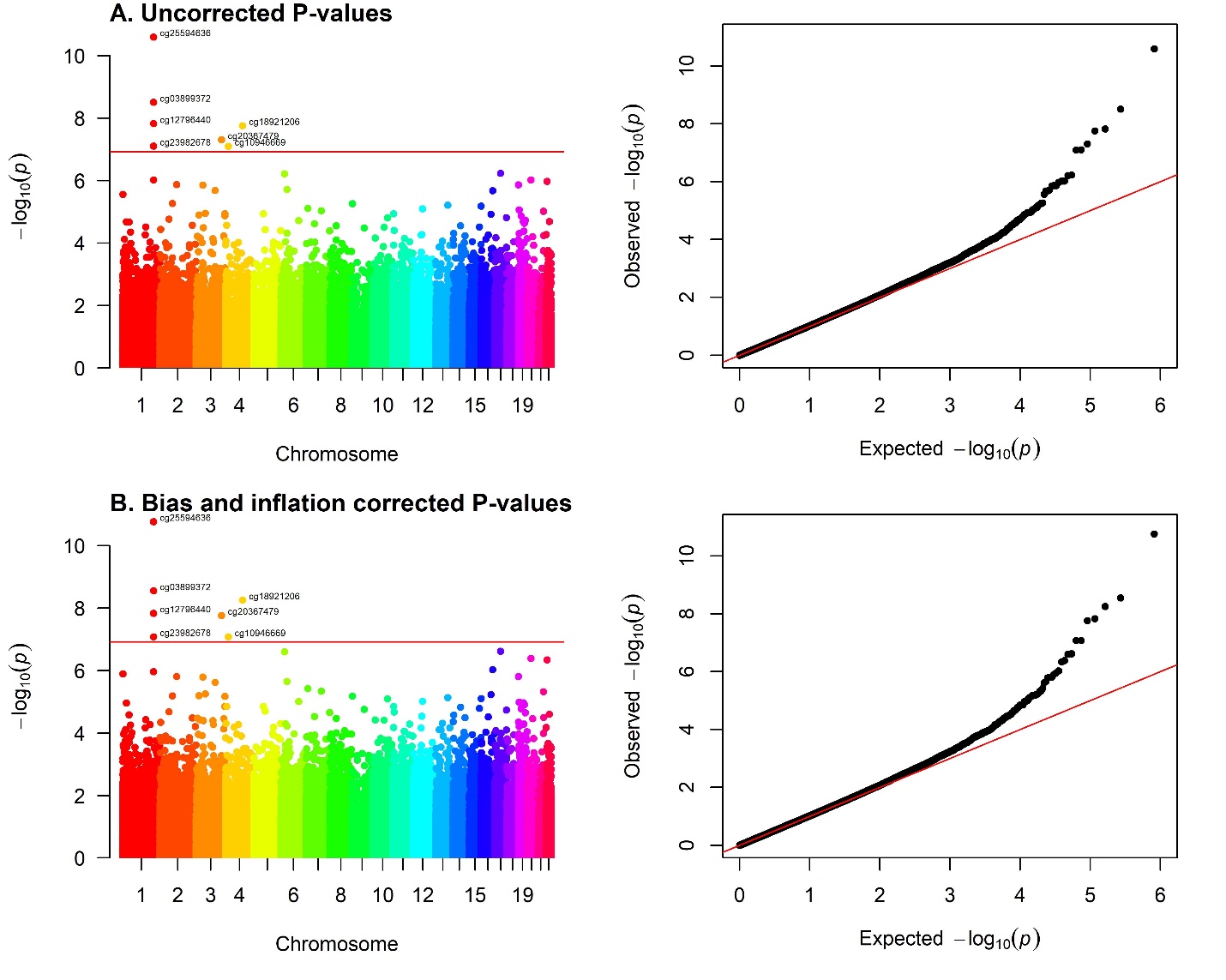
**

**Figure S3. Manhattan and QQ-Plots EWAS on clinical diagnosis of MDD (A) before and (B) after correction for bias and inflation** **using the empirical null distribution.** Adjusted for age at death, sex, PMI, neuron proportion and the first three principal components from the genotype data. Bonferroni threshold: 1.22 x 10^-07^.

| **Table S3.** Associations between DNA methylation and MDD adjusted for a polygenic risk score for MDD (calculated with PRSice ^1^ and UK Biobank summary statistics from ^2^ with a p-value < 0.05) to test if our EWAS findings were independent of genetic risk for MDD. | | | | | | | |
| --- | --- | --- | --- | --- | --- | --- | --- |
|  |  |  |  | **Main model** | | **Additionally adjusted for PRS** | |
| **cpg** | **chr** | **position** | **Nearest Gene** | **Δ beta** | **p-value** | **Δ beta** | **p-value** |
| cg25594636 | 1 | 207224388 | *YOD1* | 0.013 | 2.55E-11 | 0.013 | 2.57E-11 |
| cg03899372 | 1 | 207224102 | *YOD1* | 0.020 | 3.12E-09 | 0.020 | 3.05E-09 |
| cg12796440 | 1 | 207224331 | *YOD1* | 0.022 | 1.51E-08 | 0.022 | 1.57E-08 |
| cg18921206 | 4 | 115320920 | *UGT8* | -0.067 | 1.75E-08 | -0.067 | 1.65E-08 |
| cg20367479 | 3 | 171873675 | *FNDC3B* | -0.032 | 4.97E-08 | -0.032 | 4.91E-08 |
| cg23982678 | 1 | 207224227 | *YOD1* | 0.021 | 7.94E-08 | 0.021 | 7.76E-08 |
| cg10946669 | 4 | 20253130 | *SLIT2* | 0.013 | 8.01E-08 | 0.013 | 8.24E-08 |
| Bonferroni threshold: 1.22 x 10^-07^. Adjusted for age at death, sex, PMI, neuron proportions and the first three principal components from the genotype data. **^#^**adjusted linear regression with p-value from 1,000,000 permutations  Δ beta: This coefficient represents the mean difference of DNA methylation beta values between participants with and without MDD. Negative coefficients refer to smaller mean DNA methylation beta values in participants with MDD and positive coefficients refer to larger mean DNA methylation beta values in participants with MDD. | | | | | | | |

| **Table S4.** Replication of the significant associations with MDD from ROS/MAP in cell type-specific EWAS of MDD from Chan et al. (2020)^3^. | | | | | | | | | | | |
| --- | --- | --- | --- | --- | --- | --- | --- | --- | --- | --- | --- |
|  |  |  |  | **ROS/MAP^a^** | | **Replication**  **Bulk**^3^ | | **Replication**  **Glia**^3^ | | **Replication**  **Neurons**^3^ | |
| **cpg** | **chr** | **position** | **Nearest Gene** | **Δ beta** | **p-value** | **Δ beta** | **p-value** | **Δ beta** | **p-value** | **Δ beta** | **p-value** |
| cg25594636 | 1 | 207224388 | *YOD1* | 0.013 | 2.55E-11 | NA | NA | NA | NA | NA | NA |
| cg03899372 | 1 | 207224102 | *YOD1* | 0.020 | 3.12E-09 | NA | NA | NA | NA | NA | NA |
| cg12796440 | 1 | 207224331 | *YOD1* | 0.022 | 1.51E-08 | NA | NA | NA | NA | NA | NA |
| cg18921206 | 4 | 115320920 | *UGT8* | -0.067 | 1.75E-08 | -0.022 | 0.777 | 0.060 | 0.445 | -0.070 | 0.367 |
| cg20367479 | 3 | 171873675 | *FNDC3B* | -0.032 | 4.97E-08 | 0.176 | 0.022 | 0.079 | 0.313 | -0.013 | 0.871 |
| cg23982678 | 1 | 207224227 | *YOD1* | 0.021 | 7.94E-08 | NA | NA | NA | NA | NA | NA |
| cg10946669 | 4 | 20253130 | *SLIT2* | 0.013 | 8.01E-08 | NA | NA | NA | NA | NA | NA |
| ^a^Adjusted for age at death, sex, PMI, neuron proportions and the first three principal components from the genotype data.  Δ beta: This coefficient represents the mean difference of DNA methylation beta values between participants with and without MDD. Negative coefficientsO refer to smaller mean DNA methylation beta values in participants with MDD and positive coefficients refer to larger mean DNA methylation beta values in participants with MDD.  NA: The CpG sites from the *YOD1* and *SLIT2* loci were not available in Chan et al. (2020) ^3^. | | | | | | | | | | | |

| **Table S5.** DMRs in relation to late-life MDD without dementia. | | | | | | |
| --- | --- | --- | --- | --- | --- | --- |
| **Chr** | **Start** | **End** | **# CpGs** | **Max Δ beta^a^** | **p-value^b^** | **Genes^c^** |
| chr1 | 207224090 | 207224227 | 3 | 0.021 | 5.06E-10 | *YOD1-002, YOD1-201, PFKFB2-201* |
| Δ beta: This coefficient represents the mean difference of DNA methylation beta values between participants with and without MDD. Negative coefficients refer to smaller mean DNA methylation beta values in participants with MDD and positive coefficients refer to larger mean DNA methylation beta values in participants with MDD.  ^a^ Maximum Δ beta for the region; ^b^ Minimum FDR p-value for the region; ^c^ Annotated genes in the region | | | | | | |

**
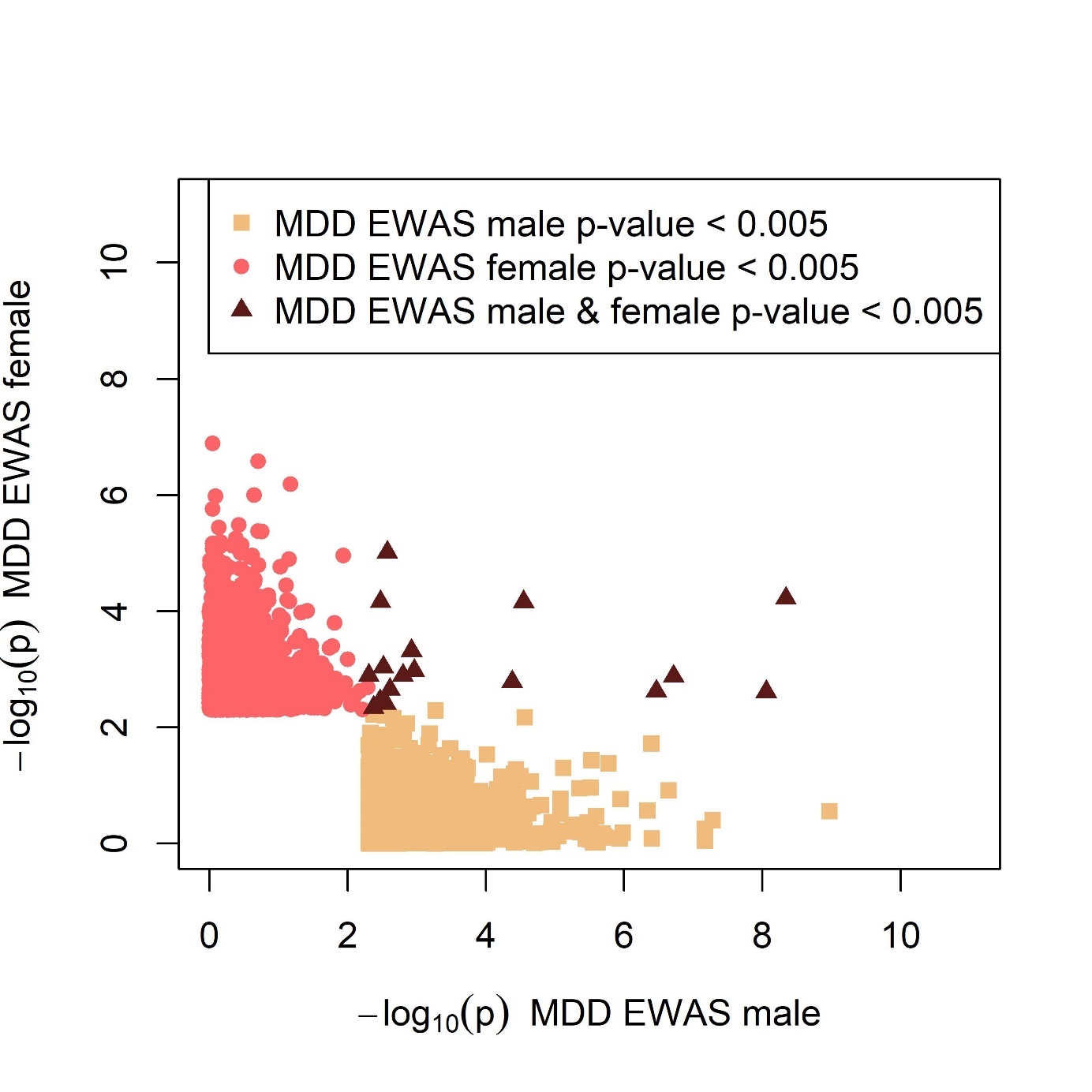
**

**Figure S4. There is some overlap between MDD-associated differentially methylated positions in male and female.** Shown is a scatterplot comparing probe-wise significance in the MDD EWAS of male (x-axis) and female (y-axis). Data are presented for CpG sites with p-values < 0.005 in the MDD EWAS of male, female or both.

| **Table S6. Pathway analysis.** Top 10 significant gene ontology terms derived from pathway analysis using missMethyl method based on the top 1000 CpGs associated with depression (dementia cases excluded). | | | | | |
| --- | --- | --- | --- | --- | --- |
|  | **TERM** | **N** | **DE** | **P.DE** | **FDR** |
| GO:0033192 | calmodulin-dependent protein phosphatase activity | 5 | 4 | 6.00E-05 | 1 |
| GO:0006783 | heme biosynthetic process | 21 | 5 | 0.0018 | 1 |
| GO:1990167 | protein K27-linked deubiquitination | 3 | 2 | 0.0030 | 1 |
| GO:0071874 | cellular response to norepinephrine stimulus | 2 | 2 | 0.0039 | 1 |
| GO:0060623 | regulation of chromosome condensation | 2 | 2 | 0.0041 | 1 |
| GO:1905145 | cellular response to acetylcholine | 2 | 2 | 0.0046 | 1 |
| GO:0051792 | medium-chain fatty acid biosynthetic process | 3 | 2 | 0.0049 | 1 |
| GO:0048669 | collateral sprouting in absence of injury | 2 | 2 | 0.0049 | 1 |
| GO:0031369 | translation initiation factor binding | 15 | 4 | 0.0050 | 1 |
| GO:0030131 | clathrin adaptor complex | 8 | 3 | 0.0052 | 1 |
| N: Number of genes in the GO term; DE: number of genes that are differentially methylated; P.DE: p-value for over-representation of the gene set; FDR: False discovery rate | | | | | |

| **Table S7.** Genes that belong to the most significant pathway from Table S5 (GO:0033192, <http://www.informatics.jax.org/go/term/GO:0033192>) | | | |
| --- | --- | --- | --- |
| **MGI Gene/Marker ID** | **Symbol** | **Name** | **Chr** |
| MGI:99878 | *PPM1A* | protein phosphatase 1A, magnesium dependent, alpha isoform | 12 |
| MGI:1918464 | *PPM1F* | protein phosphatase 1F (PP2C domain containing) | 16 |
| MGI:107164 | *PPP3CA* | protein phosphatase 3, catalytic subunit, alpha isoform | 3 |
| MGI:107164 | *PPP3CA* | protein phosphatase 3, catalytic subunit, alpha isoform | 3 |
| MGI:107164 | *PPP3CA* | protein phosphatase 3, catalytic subunit, alpha isoform | 3 |
| MGI:107164 | *PPP3CA* | protein phosphatase 3, catalytic subunit, alpha isoform | 3 |
| MGI:107163 | *PPP3CB* | protein phosphatase 3, catalytic subunit, beta isoform | 14 |
| MGI:107163 | *PPP3CB* | protein phosphatase 3, catalytic subunit, beta isoform | 14 |
| MGI:107163 | *PPP3CB* | protein phosphatase 3, catalytic subunit, beta isoform | 14 |
| MGI:107162 | *PPP3CC* | protein phosphatase 3, catalytic subunit, gamma isoform | 14 |
| MGI:107162 | *PPP3CC* | protein phosphatase 3, catalytic subunit, gamma isoform | 14 |

**
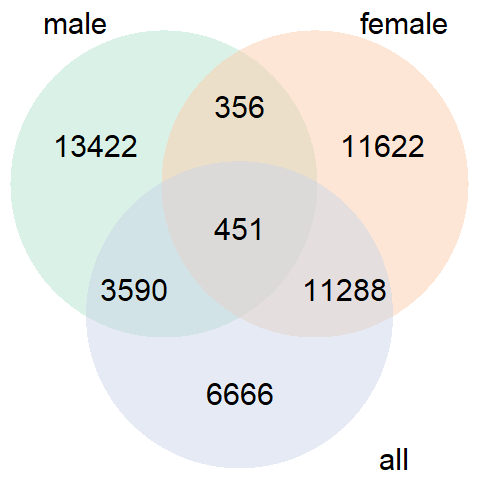
**

**Figure S5.** Overlap of nominally significant associations (p-values < 0.05) DNA methylation and MDD status in males, females and the combined dataset (all).

| **Table S8. Pathway analysis.** Top 10 significant gene ontology terms derived from pathway analysis using missMethyl method based on the 807 CpG sites that reached at least nominal significance in both sex-specific EWAS. | | | | | |
| --- | --- | --- | --- | --- | --- |
|  | **TERM** | **N** | **DE** | **P.DE** | **FDR** |
| GO:0033192 | calmodulin-dependent protein phosphatase activity | 5 | 4 | 6.00E-05 | 1 |
| GO:0006783 | heme biosynthetic process | 21 | 5 | 0.0018 | 1 |
| GO:1990167 | protein K27-linked deubiquitination | 3 | 2 | 0.0030 | 1 |
| GO:0071874 | cellular response to norepinephrine stimulus | 2 | 2 | 0.0039 | 1 |
| GO:0060623 | regulation of chromosome condensation | 2 | 2 | 0.0041 | 1 |
| GO:1905145 | cellular response to acetylcholine | 2 | 2 | 0.0046 | 1 |
| GO:0051792 | medium-chain fatty acid biosynthetic process | 3 | 2 | 0.0049 | 1 |
| GO:0048669 | collateral sprouting in absence of injury | 2 | 2 | 0.0049 | 1 |
| GO:0031369 | translation initiation factor binding | 15 | 4 | 0.0050 | 1 |
| GO:0030131 | clathrin adaptor complex | 8 | 3 | 0.0052 | 1 |
| N: Number of genes in the GO term; DE: number of genes that are differentially methylated; P.DE: p-value for over-representation of the gene set; FDR: False discovery rate | | | | | |

**
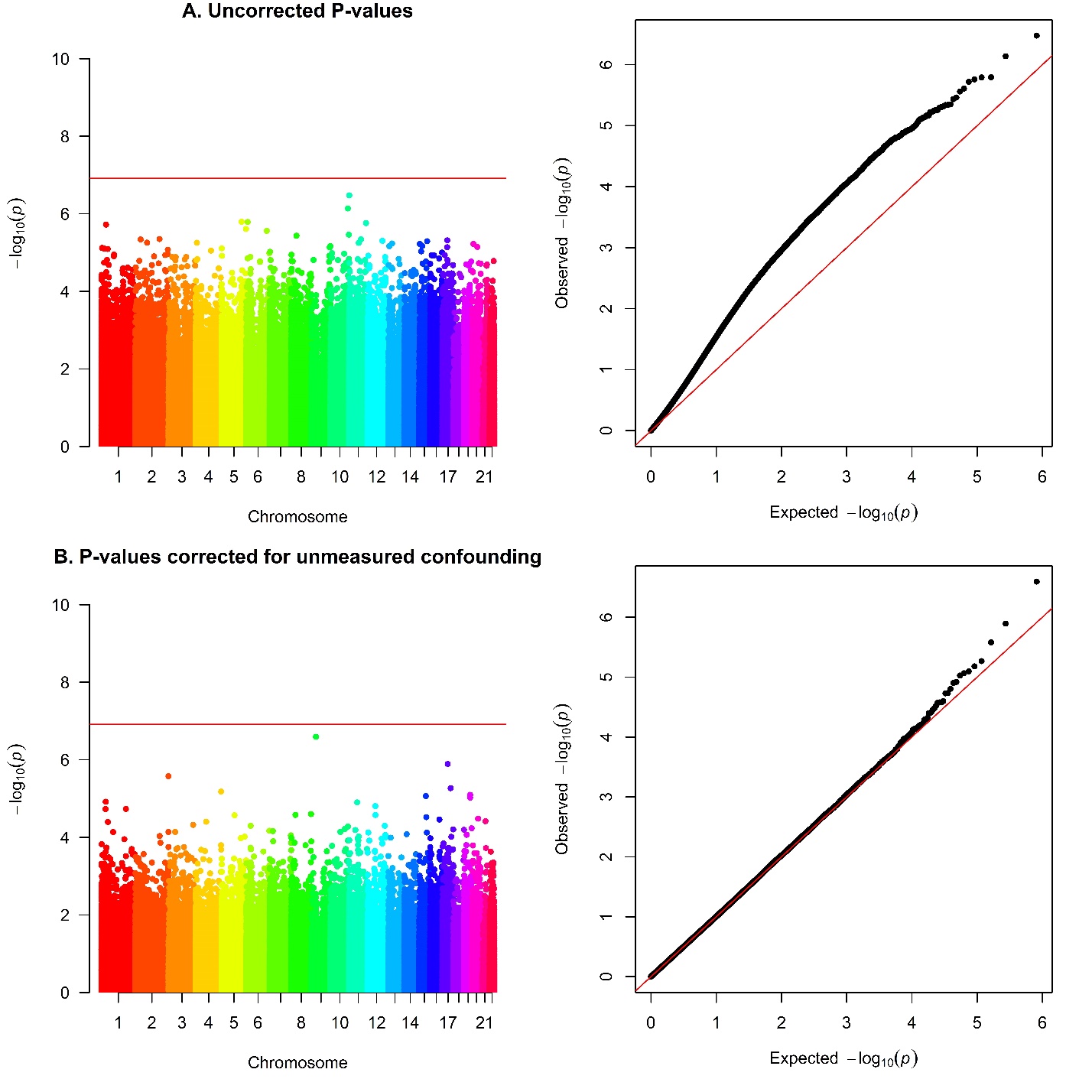
**

**Figure S6.** Manhattan and QQ-Plots for the EWAS of the polygenic risk score for MDD (calculated with PRSice ^1^ and UK Biobank summary statistics from ^2^ with a p-value < 0.05) (A) before and (B) after correction for unmeasured confounding using the R package ‘cate’. (A) and (B) were adjusted for age at death, sex, PMI, neuron proportion and the first three principal components from the genotype data. Bonferroni threshold: 1.22 x 10^-07^.

**
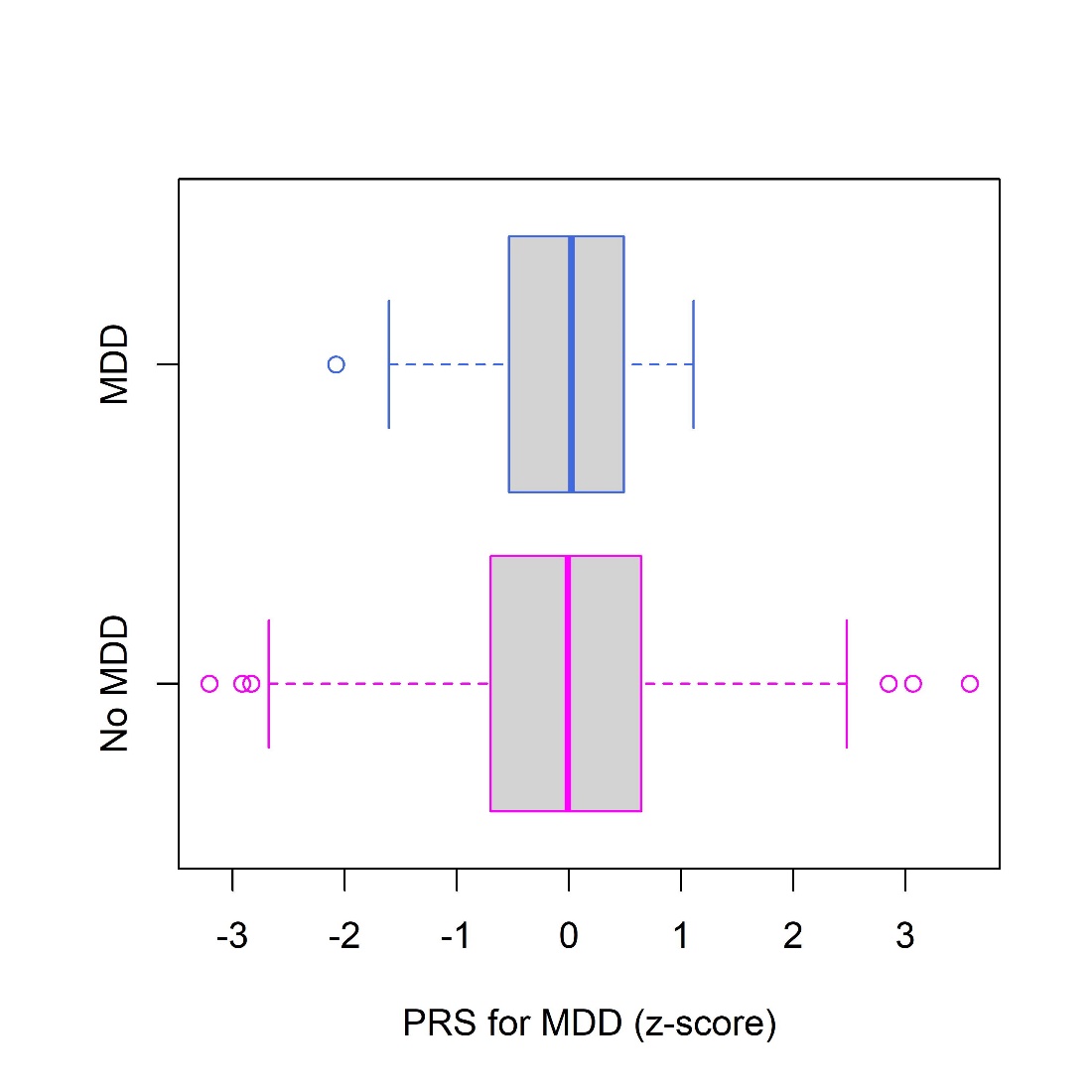
**

**Figure S7.** Distribution of the polygenic risk score for MDD (calculated with PRSice ^1^ and UK Biobank summary statistics from ^2^ with a p-value < 0.05) stratified by diagnosis of MDD at baseline visit.

**
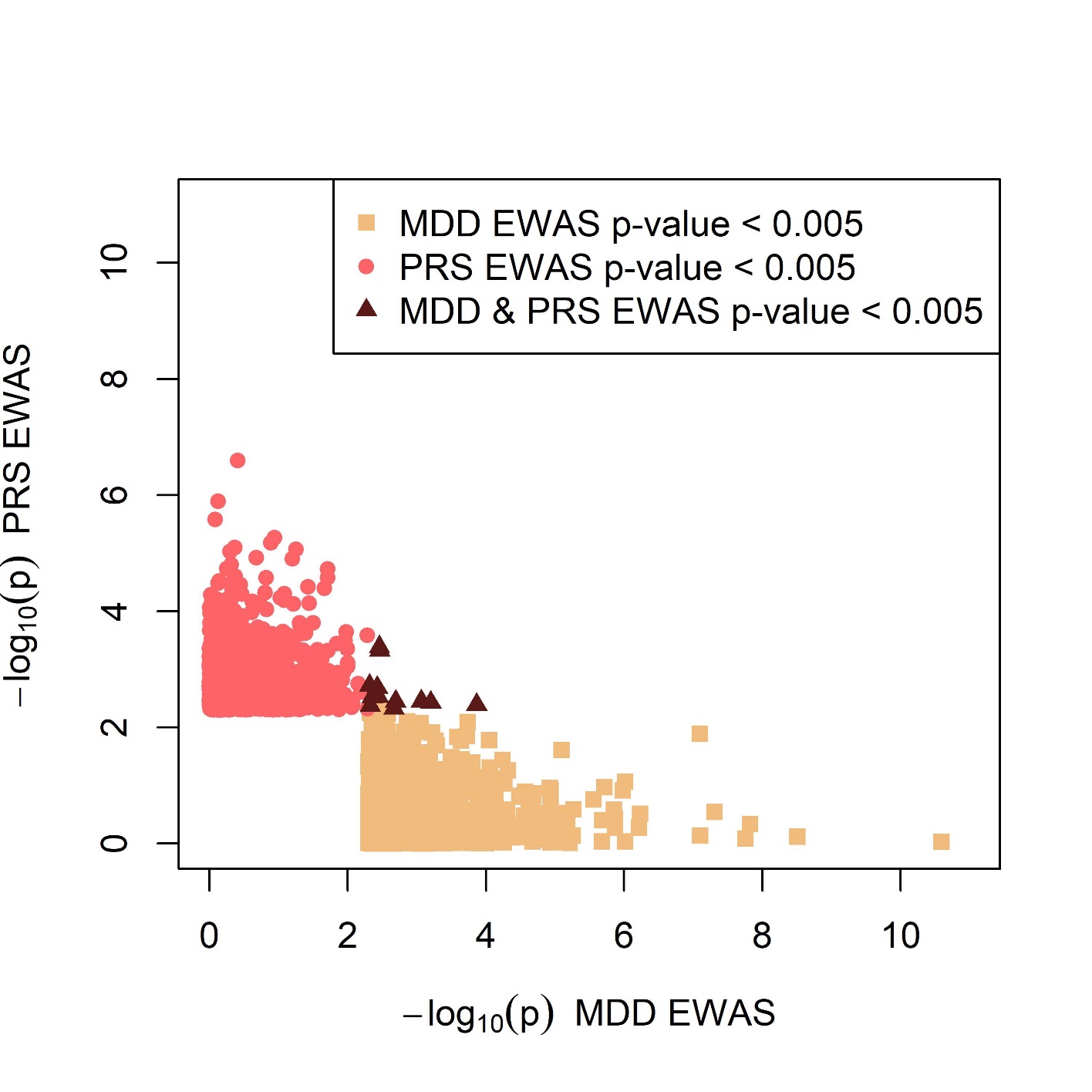
**

**Figure S8.** There is minimal overlap between nominally significant MDD-associated differentially methylated positions and those associated with the polygenic risk score for MDD (calculated with PRSice ^1^ and UK Biobank summary statistics from ^2^ with a p-value < 0.05). Shown is a scatterplot comparing probe-wise significance in the EWAS of MDD (x-axis) and PRS (y-axis). Data are presented for CpG sites with p-values < 0.005 in the MDD EWAS, PRS EWAS or both.

| **Table S10.** Replication of epigenome-wide significant hits from Story Jovanova et al. (2018) ^4^. | | | | | |
| --- | --- | --- | --- | --- | --- |
|  |  |  |  | **Story Jovanova et al. (2018)** | **ROSMAP** |
| **CpG** | **Chromosome** | **Location** | **Gene_Symbol** | **P-vale depressive symptoms** | **P-value MDD** |
| cg04987734 | 14 | 103415873 | *CDC42BPB* | 1.57*10^-08 | 0.784353 |
| cg07012687 | 17 | 80195180 | *SLC16A3* | 4.45*10^-06 | 0.960606 |
| cg08796240 | 16 | 70733832 | *VAC14* | 1.80*10^-06 | 0.890166 |
| cg06096336 | 2 | 231989800 | *PSMD1;HTR2B* | 2.51*10^-06 | 0.111201 |
| cg16745930 | 10 | 100220809 | *HPSE2* | 6.26*10^-06 | 0.423422 |
| cg09849319 | 5 | 1494983 | *LPCAT1* | 1.04*10^-04 | 0.415657 |
| cg17237086 | 22 | 40814966 | *MKL1* | 6.10*10^-06 | 0.982013 |
| cg03985718 | 2 | 105924245 | *TGFBRAP1* | 6.53*10^-05 | 0.493575 |
| cg21098005 | 20 | 44538605 | *PLTP* | 1.01*10^-04 | 0.360175 |
| cg16466652 | 19 | 6271960 | *MLLT1* | 1.57*10^-05 | 0.397096 |
| cg07884764 | 11 | 64107517 | *CCDC88B* | 1.25*10^-04 | 0.799151 |
| cg01541347 | 7 | 4729920 | *FOXK1* | 8.46*10^-04 | 0.857993 |
| cg02341197 | 21 | 34185927 | *C21orf62* | 6.80*10^-06 | 0.101789 |
| cg01947751 | 3 | 196728969 | *Intergenic* | 3.68*10^-04 | 0.752088 |
| cg13747876 | 17 | 80195402 | *SLC16A3* | 2.93*10^-06 | 0.181893 |
| cg12764201 | 1 | 105101123 | *CORT;APITD1* | 7.29*10^-05 | 0.118466 |
| cg08295111 | 5 | 133866097 | *PHF15* | 5.64*10^-04 | 0.152849 |
| cg18030453 | 3 | 45506216 | *LARS2* | 1.20*10^-07 | 0.160292 |
| cg12325605 | 3 | 56810151 | *ARHGEF3* | 5.24*10^-09 | 0.749641 |
| cg23282441 | 10 | 73533927 | *C10orf54;CDH23* | 8.63*10^-06 | 0.359098 |

| **Table S11.** Replication of epigenome-wide significant hits from Starnawska et al. (2019) ^5^. | | | | | | | |
| --- | --- | --- | --- | --- | --- | --- | --- |
|  |  |  |  | **Depressive symptoms in** ^5^ | | **MDD in ROSMAP** | |
| **Model** | **Probe_ID** | **Position** | **Gene** | **Estimate** | **P-value** | **Estimate** | **P-value** |
| Paired twin model | cg01859717 | chr6:32088654 | *ATF6B* | -0.027 | 9.87E-06 | 0.005 | 0.132 |
| Paired twin model | cg01919885 | chr4:3365330 | *RGS12* | 0.029 | 2.36E-06 | -0.006 | 0.301 |
| Paired twin model | cg02286193 | chr14:76823128 | *NA* | 0.031 | 1.01E-06 | NA | NA |
| Paired twin model | cg05777061 | chr19:51505001 | *KLK8* | -0.057 | 4.70E-07 | -0.012 | 0.425 |
| Paired twin model | cg10100767 | chr14:105246561 | *AKT1* | 0.03 | 4.47E-06 | 0.001 | 0.509 |
| Paired twin model | cg10778249 | chr19:48674746 | *LIG1* | -0.021 | 3.75E-06 | 0.004 | 0.663 |
| Paired twin model | cg12836280 | chr5:50260240 | *NA* | -0.031 | 8.01E-06 | -0.001 | 0.795 |
| Paired twin model | cg15022049 | chr11:66137145 | *SLC29A2* | -0.021 | 6.15E-06 | 0.001 | 0.878 |
| Paired twin model | cg16135936 | chr14:98629292 | *NA* | 0.035 | 8.18E-06 | -0.009 | 0.250 |
| Paired twin model | cg17350432 | chr4:841569 | *NA* | -0.016 | 7.47E-06 | 0.000 | 0.984 |
| Paired twin model | cg20250722 | chr6:26522136 | *HCG11* | -0.018 | 6.64E-06 | -0.002 | 0.834 |
| Paired twin model | cg20556803 | chr7:2114593 | *MAD1L1* | 0.03 | 5.16E-06 | 0.011 | 0.108 |
| Paired twin model | cg26241863 | chr8:145849419 | *NA* | -0.028 | 5.93E-06 | -0.002 | 0.715 |
| Unpaired twin model | cg00554948 | chr12:51631858 | *DAZAP2* | -0.016 | 3.13E-08 | 0.000 | 0.969 |
| Unpaired twin model | cg01971269 | chr5:162993061 | *NA* | -0.008 | 9.10E-06 | 0.001 | 0.512 |
| Unpaired twin model | cg03550773 | chr14:35163458 | *NA* | 0.011 | 4.20E-06 | 0.001 | 0.818 |
| Unpaired twin model | cg23050873 | chr2:234184376 | *ATG16L1* | -0.009 | 5.40E-06 | -0.004 | 0.300 |
| Unpaired twin model | cg25104234 | chr2:52281777 | *NA* | -0.014 | 8.40E-06 | -0.009 | 0.158 |
| Unpaired twin model | cg26603050 | chr1:22938172 | *NA* | 0.016 | 7.90E-06 | 0.008 | 0.268 |

**References**

1 Euesden J, Lewis CM, O’Reilly PF. PRSice: Polygenic Risk Score software. *Bioinformatics* 2015; **31**: 1466–1468.

2 Howard DM, Adams MJ, Clarke TK, Hafferty JD, Gibson J, Shirali M *et al.* Genome-wide meta-analysis of depression identifies 102 independent variants and highlights the importance of the prefrontal brain regions. *Nat Neurosci* 2019; **22**: 343–352.

3 Chan RF, Turecki G, Shabalin AA, Guintivano J, Zhao M, Xie LY *et al.* Cell Type–Specific Methylome-wide Association Studies Implicate Neurotrophin and Innate Immune Signaling in Major Depressive Disorder. *Biol Psychiatry* 2020; **87**: 431–442.

4 Jovanova OS, Nedeljkovic I, Spieler D, Walker RM, Liu C, Luciano M *et al.* DNA methylation signatures of depressive symptoms in middle-aged and elderly persons: Meta-analysis of multiethnic epigenome-wide studies. *JAMA Psychiatry* 2018; **75**: 949–959.

5 Starnawska A, Tan Q, Soerensen M, McGue M, Mors O, Børglum AD *et al.* Epigenome-wide association study of depression symptomatology in elderly monozygotic twins. *Transl Psychiatry* 2019; **9**. doi:10.1038/s41398-019-0548-9.
